# Supplementary material for: Anti-Nogo-A NG101 treatment induces changes in spinal cord micro- and macrostructure following spinal cord injury
Source: Nat Commun. 2026 May 12;17:4197. doi: 10.1038/s41467-026-71412-0 (PMC13168246; doi:10.1038/s41467-026-71412-0)
Supplement: Supplementary file 1 — Supplementary Information [file 41467_2026_71412_MOESM1_ESM.pdf]

Supporting Information for Anti-Nogo-A NG101 treatment induces changes in spinal cord micro- and macrostructure following spinal cord injury, Lynn Farner et al.

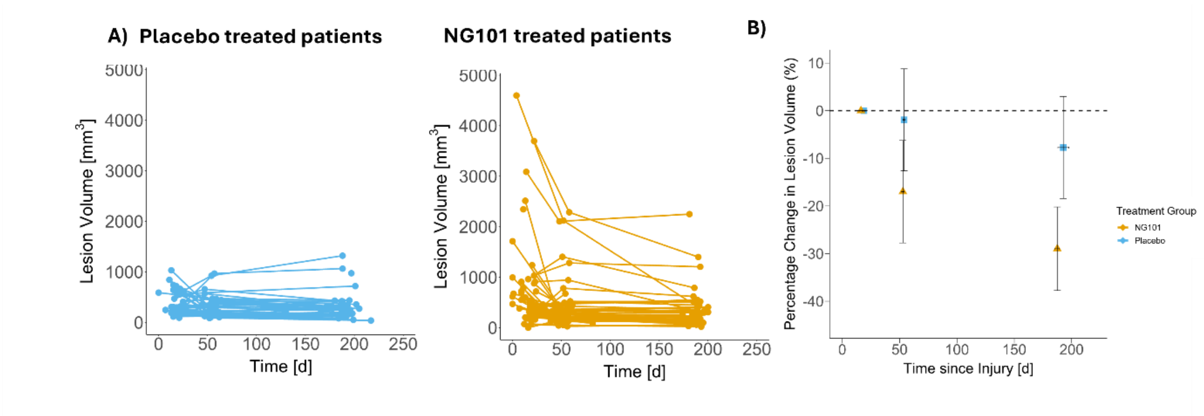

Supplementary Figure 1. Longitudinal changes in spinal cord lesion volume. A) Individual trajectories of absolute lesion volume over time (days since injury) for biologically independent placebo-treated (blue circles; n = 43) and Verum-treated (orange circles; n = 63) participants. B) Percentage change of lesion volume over time relative to the measurements at the baseline screening visit. Data are presented as mean values ± standard error of the mean (SEM) for both the percentage change in lesion volume (vertical error bars) and the time since injury (horizontal error bars) for the Verum (orange triangles) and placebo (sky blue squares) groups. Total n = 106 biologically independent participants (Verum: n = 63; Placebo: n = 43). Source Data are provided as a Source Data file.

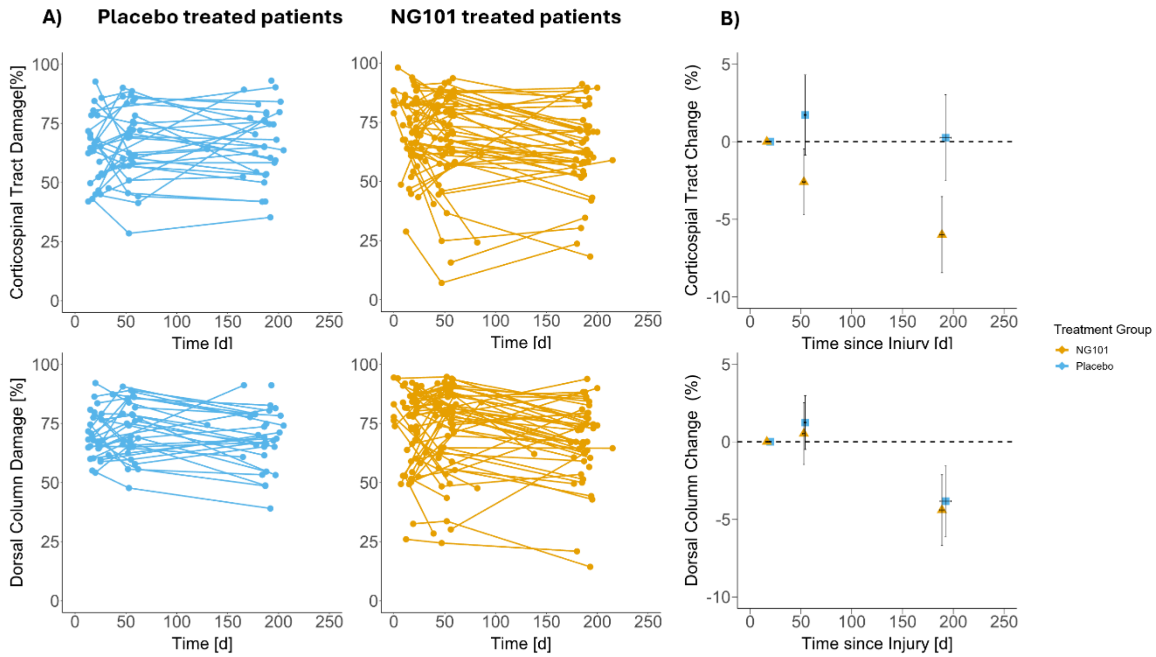

Supplementary Figure 2. Longitudinal changes in Corticospinal Tract (CST) and Dorsal Column (DC) tract damage. A) Individual trajectories of absolute tract damage in the CST and DC at the lesion level over time (days since injury) for biologically independent placebo-treated (blue circles; n = 35) and Verum-treated (orange circles; n = 60) participants. B) Percentage change of CST and DC tract damage over time relative to the baseline screening visit. Data are presented as mean values ± standard error of the mean (SEM) for both the percentage change in tract damage (vertical error bars) and the time since injury (horizontal error bars) for the Verum (orange triangles) and placebo (sky blue squares) groups. Total n = 95 biologically independent participants (Verum: n = 60; Placebo: n = 35). Abbreviations: CST = corticospinal tract; DC = dorsal column. Source Data are provided as a Source Data file.

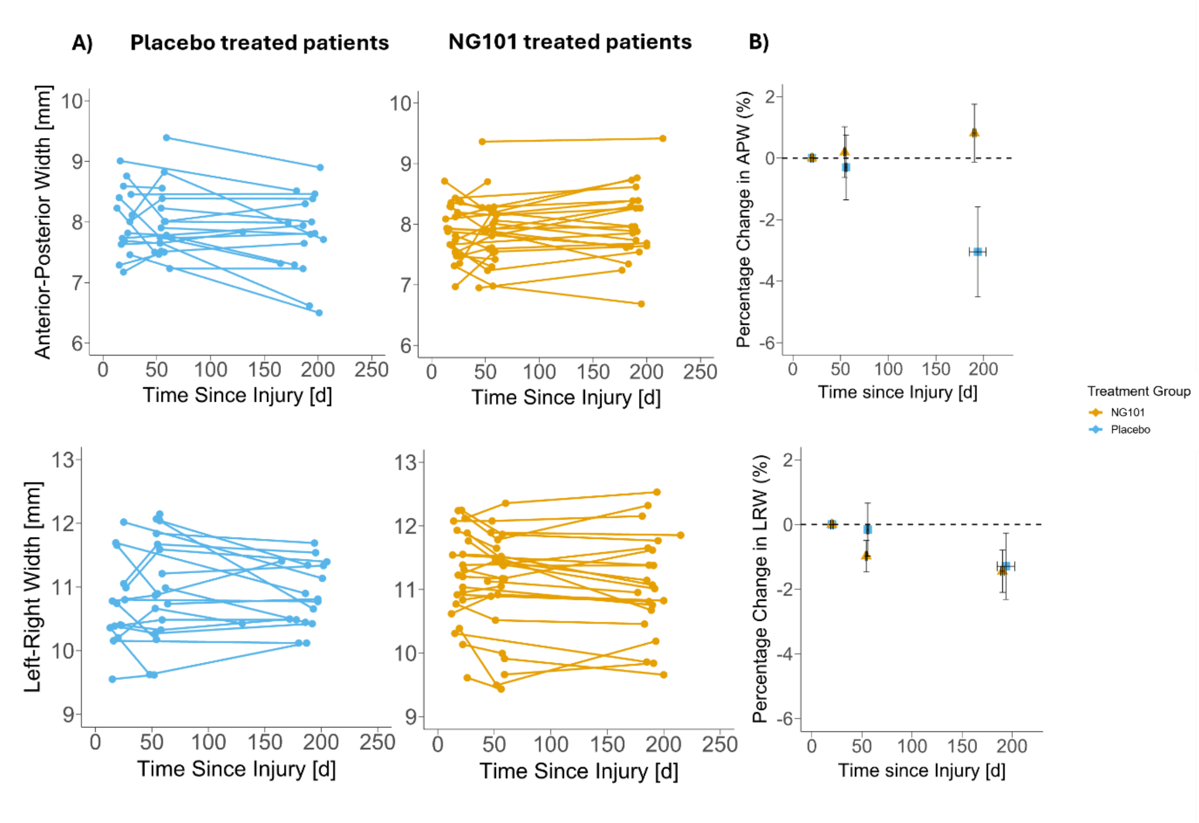

Supplementary Figure 3. Longitudinal changes in cervical spinal cord dimensions (APW and LRW). A) Individual trajectories of Anterior-Posterior Width (APW) and Left-Right Width (LRW) at the C1-C2 level over time (days since injury) for biologically independent placebo-treated (blue circles; n = 30) and Verum-treated (orange circles; n = 32) participants. B) Percentage change of APW and LRW over time relative to the measurements at the baseline screening visit. Data are presented as mean values ± standard error of the mean (SEM) for both the percentage change in dimensions (vertical error bars) and the time since injury (horizontal error bars) for the Verum (orange triangles) and placebo (blue squares) groups. Total n = 62 biologically independent participants (Verum: n = 32; Placebo: n = 30). Source Data are provided as a Source Data file.

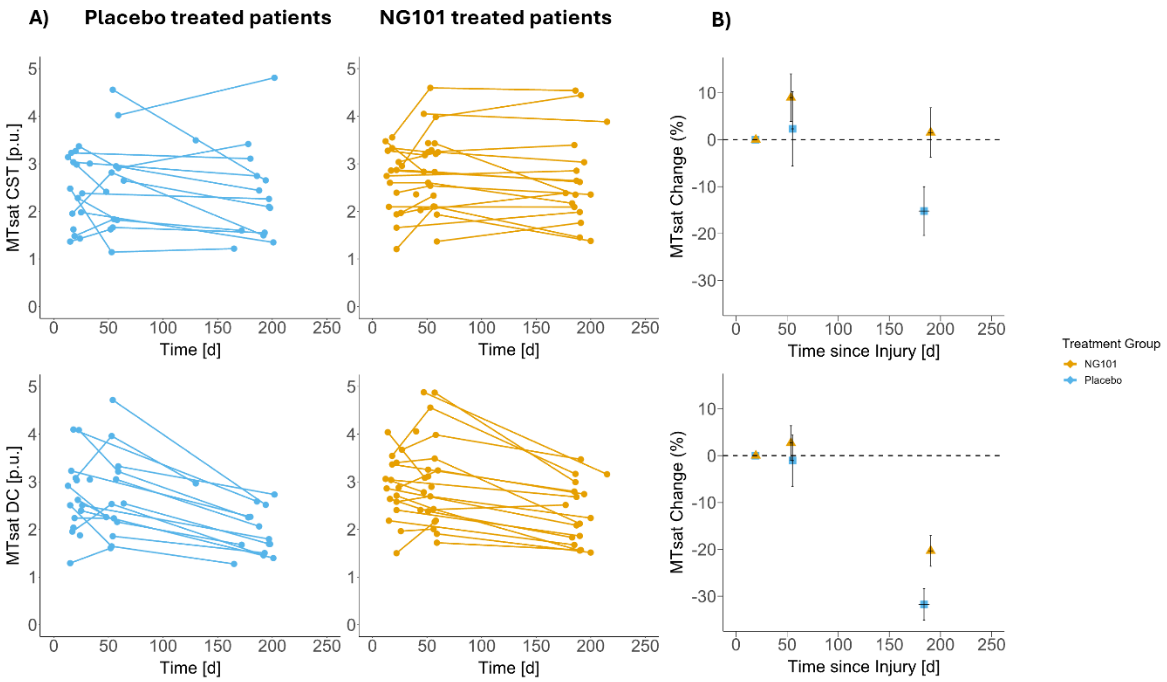

Supplementary Figure 4. Longitudinal changes in cervical spinal cord magnetization transfer saturation (MTsat). A) Individual trajectories of absolute MTsat values in the Corticospinal Tract (CST) and Dorsal Column (DC) at the C1-C2 level over time (days since injury) for biologically independent placebo-treated (blue circles; n = 22) and Verum-treated (orange circles; n = 25) participants. B) Percentage change of CST and DC MTsat over time relative to the baseline screening visit. Data are presented as mean values ± standard error of the mean (SEM) for both the percentage change in MTsat (vertical error bars) and the time since injury (horizontal error bars) for the Verum (orange triangles) and placebo (blue squares) groups. Total n = 47 biologically independent participants (Verum: n = 25 ; Placebo: n = 22). Abbreviations: CST = corticospinal tract; DC = dorsal column. Source Data are provided as a Source Data file.

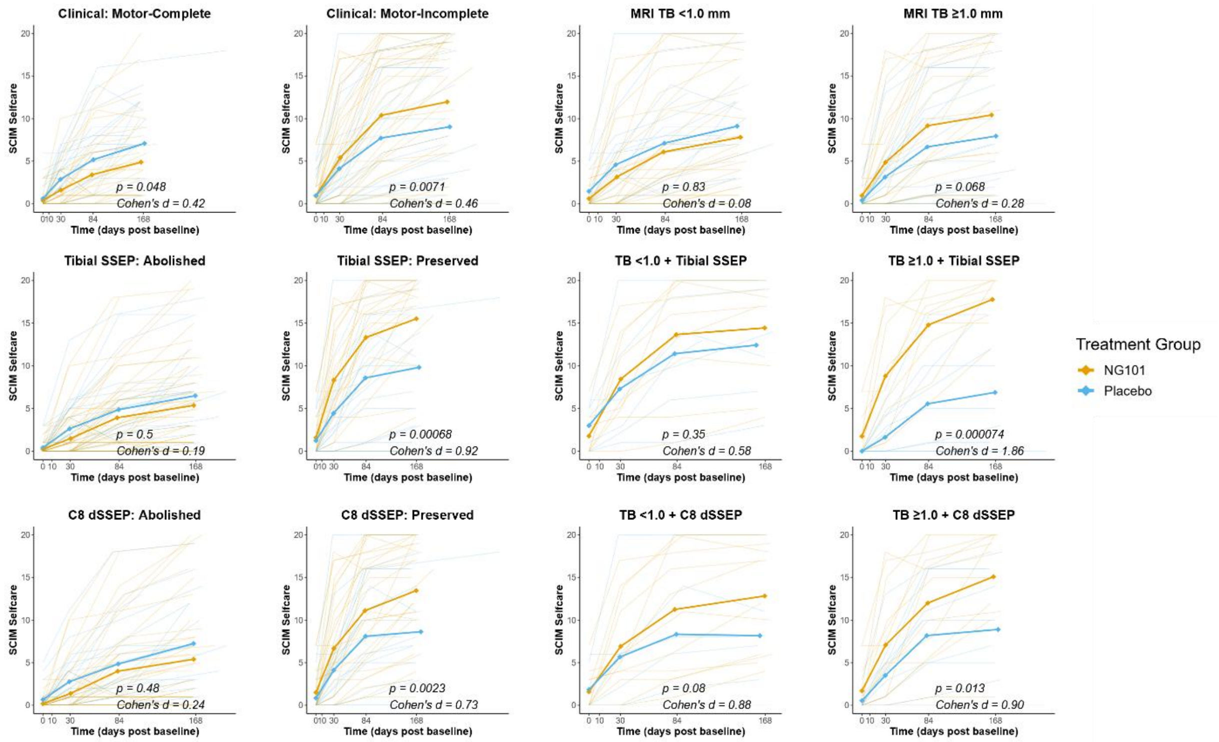

Supplementary Figure 5. Recovery trajectories of SCIM self-care across clinical, electrophysiological, and imaging stratifications. Longitudinal recovery of the Spinal Cord Independence Measure (SCIM) self-care subscore is shown over time (days post-baseline) for Verum-treated (orange) and placebo-treated (blue) participants across twelve distinct stratification groups. n represents the number of biologically independent participants in each subgroup. Data are presented as individual patient trajectories (transparent lines) and group estimated marginal means (solid lines with diamond markers). Statistical significance was assessed using a two-sided linear mixed-effects model (LMM) to evaluate the treatment-by-time interaction. No adjustments were made for multiple comparisons. For the clinical stratification, the motor-incomplete group (n=63; Verum: 38, Placebo: 25) showed t(182.46) = 2.72, p = 0.0071, while the motor-complete group (n=63; Verum: 40, Placebo: 23) showed t(185.65) = -1.99, p = 0.048. In the electrophysiological cohorts, the preserved tibial SSEP group (n=41; Verum: 24, Placebo: 17) demonstrated t(118.16) = 3.49, p = 0.00068, and the abolished tibial SSEP group (n=74; Verum: 50, Placebo: 24) showed t(217.50) = -0.68, p = 0.5. The preserved C8 dSSEP group (n=48; Verum: 28, Placebo: 20) resulted in t(143.26) = 3.10, p = 0.0023, whereas the abolished C8 dSSEP group (n=66; Verum: 45, Placebo: 21) resulted in t(188.74) = -0.71, p = 0.48. Regarding imaging stratifications, the TB ≥ 1.0 mm group (n=48; Verum: 26, Placebo: 22) showed t(136.30) = 1.84, p = 0.068, and the TB < 1.0 mm group (n=50; Verum: 33, Placebo: 17)

showed t(150.28) = -0.21, p = 0.83. For the combined stratifications, the TB ≥ 1.0 + Tibial SSEP group (n=21; Verum: 12, Placebo: 9) yielded t(59.12) = 4.29, p = 0.000074, the TB < 1.0 + Tibial SSEP group (n=16; Verum: 9, Placebo: 7) yielded t(50.18) = 0.94, p = 0.35, the TB ≥ 1.0 + C8 dSSEP group (n=24; Verum: 13, Placebo: 11) yielded t(70.98) = 2.55, p = 0.013, and the TB < 1.0 + C8 dSSEP group (n=18; Verum: 12, Placebo: 6) yielded t(56.10) = 1.78, p = 0.08. Time 0 indicates the baseline visit performed one day prior to intervention. Abbreviations: SSEP = somatosensory evoked potentials; TB = midsagittal tissue bridges. Source Data are provided as a Source Data file.

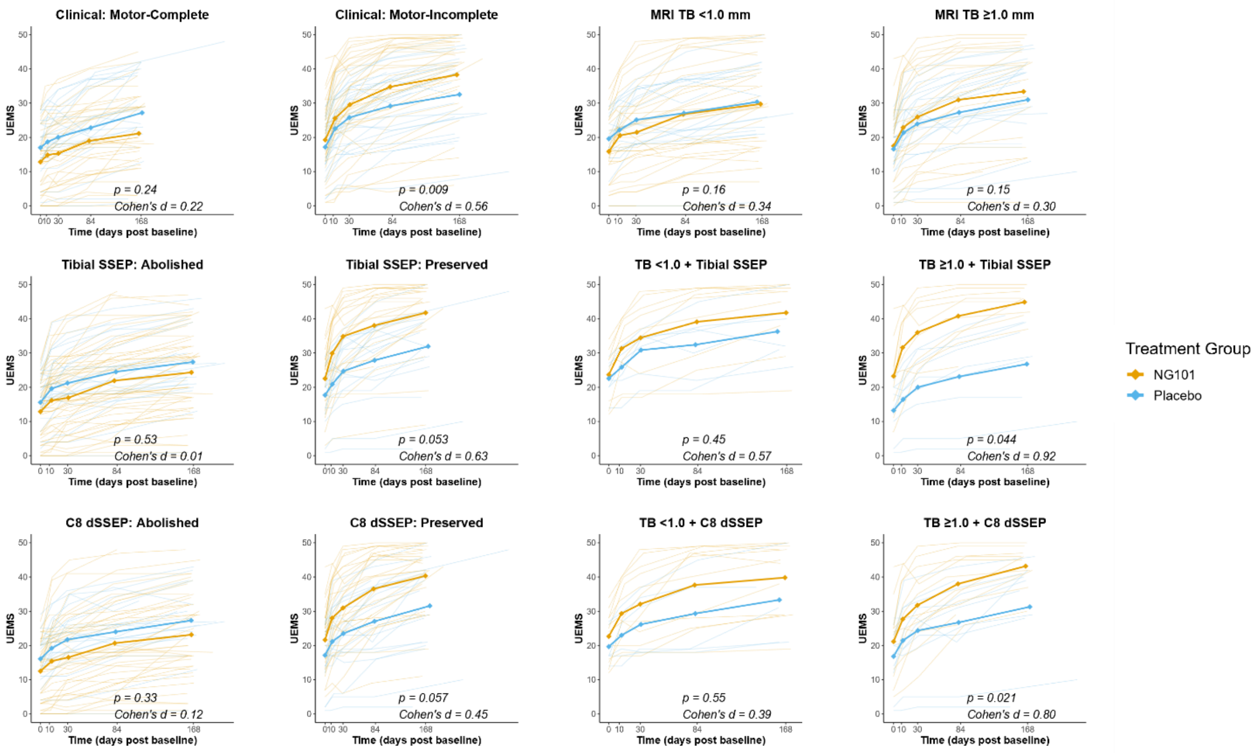

Supplementary Figure 6. Recovery trajectories of UEMS across clinical, electrophysiological, and imaging stratifications. Longitudinal recovery of the Upper Extremity Motor Score (UEMS) is shown over time (days post-baseline) for Verum-treated (orange) and placebo-treated (blue) participants across twelve distinct stratification groups. n represents the number of biologically independent participants in each subgroup. Data are presented as individual patient trajectories (transparent lines) and group estimated marginal means (solid lines with diamond markers). Statistical significance was assessed using a two-sided linear mixed-effects model (LMM) to evaluate the treatment-by-time interaction. No

adjustments were made for multiple comparisons. For the clinical stratification, the motor-incomplete group (n=63; Verum: 38, Placebo: 25) showed $t(242.62) = 2.63$, $p = 0.009$, while the motor-complete group (n=63; Verum: 40, Placebo: 23) showed $t(241.42) = -1.17$, $p = 0.24$. In the electrophysiological cohorts, the preserved tibial SSEP group (n=41; Verum: 24, Placebo: 17) demonstrated $t(155.83) = 1.95$, $p = 0.053$, and the abolished tibial SSEP group (n=74; Verum: 50, Placebo: 24) showed $t(284.68) = 0.62$, $p = 0.53$. The preserved C8 dSSEP group (n=48; Verum: 28, Placebo: 20) resulted in $t(190.47) = 1.92$, $p = 0.057$, whereas the abolished C8 dSSEP group (n=66; Verum: 45, Placebo: 21) resulted in $t(245.68) = 0.97$, $p = 0.33$. Regarding imaging stratifications, the TB ≥ 1.0 mm group (n=48; Verum: 26, Placebo: 22) showed $t(180.70) = 1.45$, $p = 0.15$, and the TB < 1.0 mm group (n=50; Verum: 33, Placebo: 17) showed $t(197.14) = 1.42$, $p = 0.16$. For the combined stratifications, the TB ≥ 1.0 + Tibial SSEP group (n=21; Verum: 12, Placebo: 9) yielded $t(77.95) = 2.06$, $p = 0.044$, the TB < 1.0 + Tibial SSEP group (n=16; Verum: 9, Placebo: 7) yielded $t(66.10) = 0.76$, $p = 0.45$, the TB ≥ 1.0 + C8 dSSEP group (n=24; Verum: 13, Placebo: 11) yielded $t(95.33) = 2.34$, $p = 0.021$, and the TB < 1.0 + C8 dSSEP group (n=18; Verum: 12, Placebo: 6) yielded $t(74.08) = 0.59$, $p = 0.55$. Time 0 indicates the baseline visit performed one day prior to intervention. Abbreviations: SSEP = somatosensory evoked potentials; TB = midsagittal tissue bridges. Source Data are provided as a Source Data file.

		Delta UEMS				Delta SCIMsc			
	Enrolment rate for NISCI Full Analysis Set (N=126)	Required total sample size for 80% power (Nreq)	Enrolment period (months)	Total pre-screening sample size (N)	Total screening sample size (N)	Required total sample size for 80% power (Nreq)	Enrolment period (months)	Total pre-screening sample size (N)	Total screening sample size (N)
**I. Clinical stratification**									
**Injury severity**									
Motor-complete	1.62	530	328	3894	1086	146	90	1073	272
Motor-incomplete	1.62	82	51	603	168	120	74	882	224
**II. Electrophysiological stratification**									
**Tibial SSEPs**									
Abolished	1.69	207068	122358	1452394	405006	690	408	4840	1227
Preserved	0.97	66	68	804	224	32	33*	392	99
**C8 dSSEPs**									
Abolished	1.69	1824	1078	12794	3568	436	258	3058	775
Preserved	1.23	126	102	1215	339	50	41	482	122
**III. Imaging stratufication**									
**Midsagittal Tissue Bridges**									
<1mm	1.28	220	172	2037	568	3642	2841	33720	8551
>=1mm	1.66	286	173	2051	572	310	187	2223	564
**reserved tibial SSEP) + Imaging Stratification**									
**Midsagittal Tissue Bridges**									
<1mm	0.41	78	190	2257	629	76	185	2199	558
>=1mm	0.54	32	59	705	197	10	27*	320	81
**reserved C8 dSSEP) + Imaging Stratification**									
**Midsagittal Tissue Bridges**									
<1mm	0.46	166	360	4269	1190	34	74	874	222
>=1mm	0.62	42	68	810	226	32	52	617	157

Enrolmend periods were estimated based on the recruitment rate in the original NISCI trial.
Total pre-screening sample sizes were estimated from the pre-screening rate from the original NISCI trial (463/39months = 11.87 participants per month).
Total screening sample sizes were estimated based on the final screening rate of the original NISCI trial (129/39months = 3.31 participants per month).

* Numbers based on actual enrolment period from the original NISCI trial.

Supplementary Table 1. Recruitment rates, enrolment periods and number of pre-screened participants for NG101 vs. placebo comparisons across stratification strategies. Upper Extremity Motor Score (UEMS) and spinal cord independence measure (SCIM) self-care subscore recovery at 6 months post-baseline (Δ6-months - baseline) are shown for five stratification approaches: (I) Clinical stratification (motor-complete vs. motor-incomplete), (II) Electrophysiological stratification using preserved vs. abolished tibial somatosensory evoked potentials (SSEP) or C8 dermatomal SSEP (dSSEP), (III) MRI-based stratification using midsagittal tissue bridge (TB) thickness (< 1.0 mm vs. ≥ 1.0 mm), (IV) Combined MRI and tibial SSEP stratification and C8 dSSEP stratification. Estimated total sample sizes (N_{req}) indicate the number of participants required to detect a treatment effect with 80% power ($\alpha = 0.05$), based on observed effect sizes and unrounded data. Enrolment periods were estimated based on the recruitment rate in the original NISCI trial. Source Data are provided as a Source Data file.

Center	Scanner Model	Field Strengh [T]	Protocol	Slice Thickness [mm]	In-plane Resolution [mm]	TR [ms]	TE [ms]	FA [°]
**BCA**	Siemens Verio	3	MPM (MT/PD/T1)	1	1.0 x 1.0	37 / 18 / 18	2.46-14.76[a]	6 / 4 / 25
			T2w Sagittal	2.75	0.34 x 0.34	4240	84	124-160
**BCM**	Siemens Skyra	1.5 / 3	MPM (MT/PD/T1)	1	1.0 x 1.0	37 / 18 / 18	2.46-14.76[a]	6 / 4 / 25
			T2w Sagittal	2.75	0.34 x 0.34	3500	84	6 / 4 / 25
**BRN**	Philips Achieva	3	T2w Sagittal	3.3	0.37 x 0.37	3500	110	90
**BSL**	Siemens Prisma	3	MPM (MT/PD/T1)	1	1.0 x 1.0	37 / 18 / 18	2.46-14.76[a]	6 / 4 / 25
			T2w Sagittal	2.75	0.25 x 0.35	3500	80	160
**BYH**	Siemens Skyra	3	MPM (MT/PD/T1)	1	1.0 x 1.0	37 / 18 / 18	2.46-14.76[a]	6 / 4 / 25
			T2w Sagittal	3.3-4.4	0.54-0.81	3150-4370	75-108	150-160
**HDG**	Siemens Verio	3	T2w Sagittal	1	1.0 x 1.0	37 / 18 / 18	2.46-14.76[a]	6 / 4 / 25
			T2w Sagittal	2.75	0.69-0.78	3500	75	160
**HLE**	Philips Ingenia	3	MPM (MT/PD/T1)	1	1.0 x 1.0	37 / 18 / 18	2.46-14.76[a]	6 / 4 / 25
			T2w Sagittal	2.0-2.75	0.37-0.78	1200-3313	80-150	90
**HLU**	Toshiba Vantage Titan	1.5	T2w Sagittal	2.7	0.34 x 0.34	1200	55	90
**MNU**	Philips Achieva	3	MPM (MT/PD/T1)	1	1.0 x 1.0	37 / 18 / 18	2.46-14.76[a]	6 / 4 / 25
			T2w Sagittal	2.75	0.44 x 0.44	3000-3216	80	90
**NTL**	Philips Achieva	3	MPM (MT/PD/T1)	1	1.0 x 1.0	37 / 18 / 18	2.46-14.76[a]	6 / 4 / 25
			T2w Sagittal	2.75	0.44 x 0.44	3000	80	90
**PRG**	Siemens Magnetom Sola	1.5	T2w Sagittal	2.75	0.34 x 0.34	3500	75	160
**ZRH**	Siemens Skyra	3	MPM (MT/PD/T1)	1	1.0 x 1.0	37 / 18 / 18	2.46-14.76[a]	6 / 4 / 25
			T2w Sagittal	2.75	0.34-0.57	3500-3760	84-91	136-160

[a] *MPM sequences utilized 6 equidistant echoes (2.46, 4.92, 7.38, 9.84, 12.30, 14.76 ms).*

Supplementary Table 2 Summary of MRI hardware and sequence specifications across the NISCI study. Quantitative Multi-Parameter Mapping (MPM) was performed according to the standardized protocol described by Leutritz et al. (2020), utilizing three 3D multi-echo FLASH sequences (MT-, PD-, and T1-weighted) harmonized across all scanner platforms.Clinical T2-weighted sagittal scans were acquired using center-specific protocols; parameter ranges for slice thickness, in-plane resolution, repetition time (TR), echo time (TE), flip angle (FA), and magnetic field strength are detailed by center. Participating centers included: Barcelona (BCA), Bochum (BCM), Berlin (BRN), Basel (BSL), Bayreuth (BYH), Heidelberg (HDG), Halle (HLE), Hessisch-Lichtenau (HLU), Murnau (MNU), Nottwil (NTL), Prague (PRG), and Zurich (ZRH).

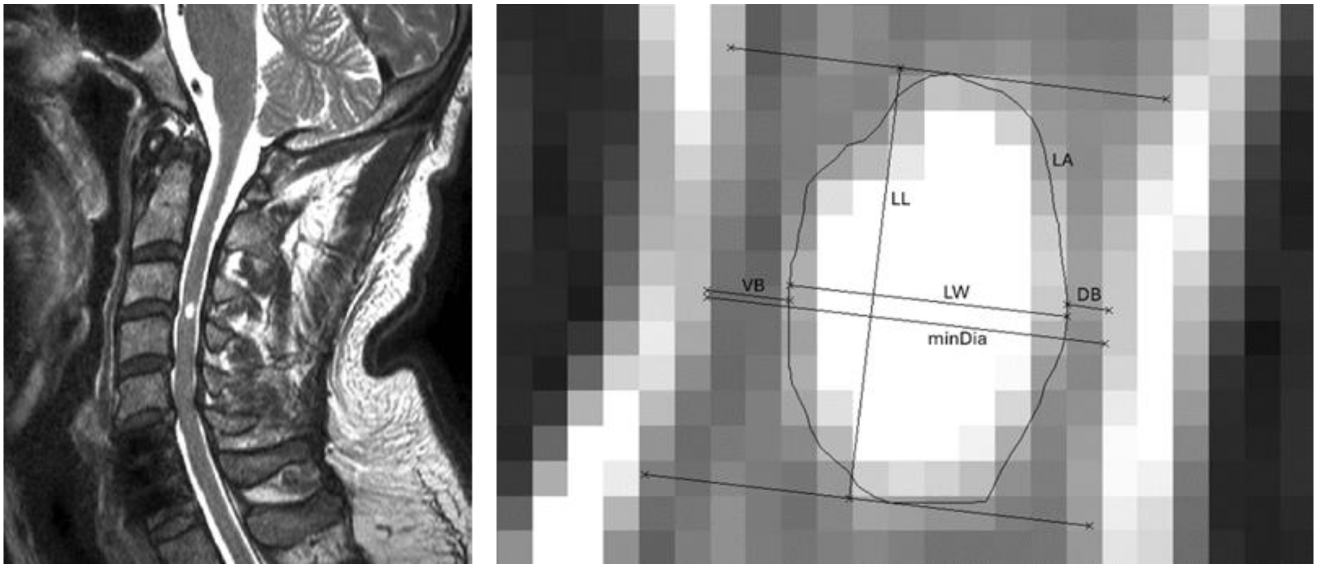

Supplementary Figure 7. Sagittal T2-weighted MRI of the spinal cord. The midsagittal slice (left) shows the lesion as a hyperintense signal. The zoomed-in view (right) highlights the lesion site with segmented parameters including lesion area (LA), width (LW), length (LL), ventral and dorsal tissue bridges (VB, DB), and minimal diameter (minDia). Segmentation was performed in JIM7.

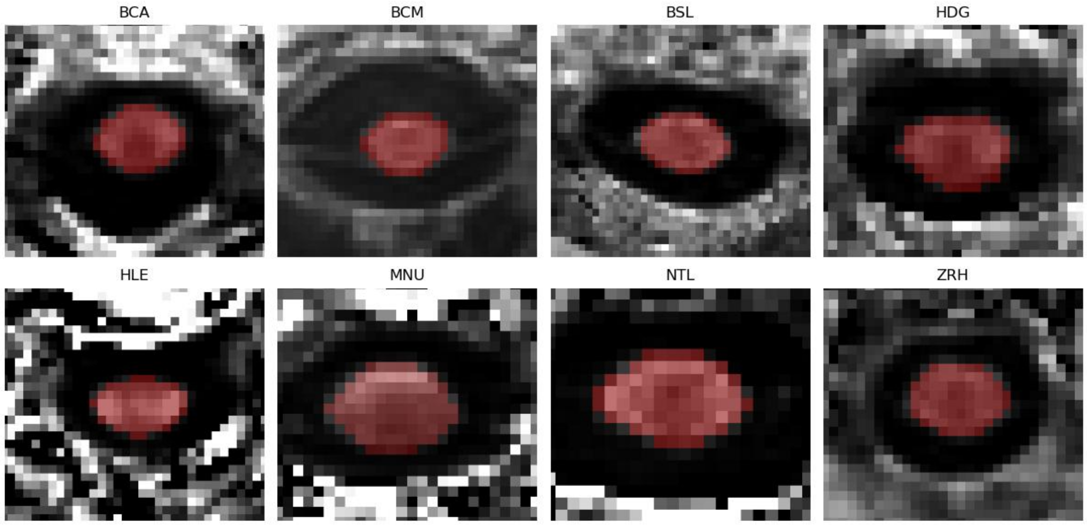

Supplementary Figure 8. Representative axial slices from all centers included in the CSA analysis. Shown are images from Barcelona (BCA), Bochum (BCM), Basel (BSL), Heidelberg (HDG), Halle (HLE), Murnau (MNU), Nottwil (NTL), and Zurich (ZRH).

Treatment	MRI Biomarker	Clinical Outcome	r[95% CI]	p-value
V	lesion_width_first	UEMS	-0.319 [-0.547, -0.048]	0.0225
V	delta_RL	UEMS	-0.532 [-0.807, -0.07]	0.0278
V	lesion_width_first	SCIM	-0.281 [-0.514, -0.009]	0.0437
V	TB_first	SCIM	0.301 [0.031, 0.531]	0.0299
P	lesion_width_first	UEMS	-0.436 [-0.666, -0.131]	0.00697
P	delta_Total_Volume	UEMS	-0.389 [-0.639, -0.064]	0.021

Supplementary Table 3. Significant Pearson correlations between structural MRI biomarkers and 6-month functional recovery. This table presents significant Pearson correlation coefficients (r) and associated 95% confidence intervals relating structural MRI biomarkers (including lesion metrics (lesion width, lesion volume, tissue bridges [TB]) and right-left width [RL]) to 6-month functional recovery outcomes, specifically the Upper Extremity Motor Score (UEMS) and Spinal Cord Independence Measure (SCIM) self-care subscore. Correlations are provided for both baseline biomarker values and 6-month changes (Δ[6 months-baseline]). Statistical significance was determined using uncorrected two-sided t-tests. Analysis populations are derived from biologically independent participants. Exact p-values and correlation strengths are listed for each significant biomarker-outcome pair. Source Data are provided as a Source Data file.

Enrolment Details

Inclusion Criteria:

1. Male or female, 18 through 70 years of age
2. Acute cervical spinal cord injury (SCI) (Neurological level of injury C1 ≤ lesion ≤ C8) with confirmed classification of ASIA impairment scale (AIS) A-D at screening and predicted upper extremities motor score (UEMS) recovery of less than 41/50 (according to the URP prediction model)
3. 4-28 days post-injury (i.e. initiation of bolus injection within 4-28 days post-injury)
4. Tetraplegic patients who are allowed to start treatment are those who either do not require mechanical ventilation or who do not completely depend on mechanical ventilation but show some degree of spontaneous ventilation. Only those modes of ventilation where the patient show active initiation of breathing are allowed (e.g. continuous positive airway pressure (CPAP))
5. Hemodynamically and clinically stable according to the acute SCI condition at baseline
6. For patients of childbearing potential, use of reliable means of contraception as described below during the treatment period and for at least six months after the last dose of study drug: Males and Females of child bearing potential, who are willing to use a highly effective method of contraception [either combined hormonal contraception associated with inhibition of ovulation (oral, intravaginal, transdermal), progestogen-only hormonal contraception associated with inhibition of ovulation (oral, injectable, implantable), intrauterine device, intrauterine hormonereleasing system, bilateral tubal occlusion, vasectomized partner or sexual abstinence)], or women not of child bearing potential, defined as women who have been surgically sterilized (total hysterectomy or bilateral oophorectomy, bilateral tubal ligation, staples, or another type of sterilization) or are postmenopausal for at least 2 years. Individuals who are convincingly sexually abstinent are also eligible. Sexual inactivity by abstinence must be consistent with the preferred and usual lifestyle of the subject. Periodic abstinence (e.g., calendar ovulation, symptothermal, or post-ovulation methods) and withdrawal are not acceptable methods of
contraception.
7. Written informed consent by patient before any study assessment is performed. If the patient is only able to consent orally a witness signs and confirms the patient's consent,
8. Cooperation and willingness to complete all aspects of the study
9. Ability of subject to understand character and individual consequences of the study

Exclusion Criteria:

1. Complete anatomical transection confirmed by magnetic resonance imaging (MRI).
2. Trauma caused by ballistic or other injury that directly penetrates the spinal cord including gunshot and knife wounds.
3. Multiple levels of clinically relevant spinal cord lesions.
4. Major brachial or lumbar plexus damage/trauma.
5. Significant head trauma (e.g. cortical damage/lesion), or other injury that was, in the opinion of the investigator, sufficient to interfere with the assessment of the spinal cord function or otherwise compromise the validity of the patient's data.
6. Other significant pre-existing or current severe systemic disease such as lung, liver (exception: history of uncomplicated Hepatitis A), gastrointestinal, cardiac, immunodeficiency

(including anamnestic known HIV) or kidney disease; or active malignancy or any other condition as determined by history or laboratory investigation that could cause a neurological deficit including syphilis, myelopathy, clinically relevant polyneuropathy, etc.

7. History of or an acute episode of Guillain-Barre syndrome.
8. History of recent (6 months) meningitis or meningoencephalitis.
9. History of refractory epilepsy.
10. Patients with uncontrolled bleeding diathesis and/or who require uninterrupted concomitant therapeutic anticoagulation (e.g. phenoprocoumon (Marcumar®), heparin/heparinoids and new oral anticoagulants) at a higher dose than for the prophylaxis of venous thromboembolism
11. Presence of any unstable medical or psychiatric condition (defined by the Diagnostic and Statistical Manual of Mental Disorders, Edition 4 (DSM-IV)) that could reasonably have been expected to subject the patient to unwarranted risk from participation in the study or result in a significant deterioration of the patient's clinical course.
12. Drug dependence (as defined by DSM-IV) any time during the 6 month’s preceding study entry.
13. Pregnant or nursing (lactating) women, where pregnancy is defined as the state of a female after conception and until the termination of gestation, confirmed by a positive human chorionic gonadotropin (hCG) laboratory test (> 5 mIU/mL).
14. History of a life-threatening allergic or immune mediated reaction.
15. Patients with the presence of infection around the location where the spinal needle insertions are planned for applying the intrathecal injections.
16. Inability to communicate effectively with the neurological examiner such that the validity of the patient's data could be compromised.
17. Participation in any clinical investigation within 4 weeks prior to dosing or longer if required by local regulations, and for any other limitation of participation based on local regulations.
18. Patients who are unconscious, including those patients who are unconscious due to medication causing marked sedation.
19. History of hypersensitivity to the investigational medicinal product or
20. to any drug with similar chemical structure.
