# Supplementary material for: Anti-Nogo-A NG101 treatment induces changes in spinal cord micro- and macrostructure following spinal cord injury
Source: Nat Commun. 2026 May 12;17:4197. doi: 10.1038/s41467-026-71412-0 (PMC13168246; doi:10.1038/s41467-026-71412-0)
Supplement: Supplementary file 2 — Reporting Summary [file 41467_2026_71412_MOESM2_ESM.pdf]

nature portfolio

Corresponding author(s): Patrick Freund

Last updated by author(s): Feb 24, 2026

Reporting Summary

Nature Portfolio wishes to improve the reproducibility of the work that we publish. This form provides structure for consistency and transparency in reporting. For further information on Nature Portfolio policies, see our Editorial Policies and the Editorial Policy Checklist.

Statistics

For all statistical analyses, confirm that the following items are present in the figure legend, table legend, main text, or Methods section.

n/a	Confirmed	
☐	☒	The exact sample size (n) for each experimental group/condition, given as a discrete number and unit of measurement
☐	☒	A statement on whether measurements were taken from distinct samples or whether the same sample was measured repeatedly
☐	☒	The statistical test(s) used AND whether they are one- or two-sided<br>*Only common tests should be described solely by name; describe more complex techniques in the Methods section.*
☐	☒	A description of all covariates tested
☐	☒	A description of any assumptions or corrections, such as tests of normality and adjustment for multiple comparisons
☐	☒	A full description of the statistical parameters including central tendency (e.g. means) or other basic estimates (e.g. regression coefficient) AND variation (e.g. standard deviation) or associated estimates of uncertainty (e.g. confidence intervals)
☐	☒	For null hypothesis testing, the test statistic (e.g. F, t, r) with confidence intervals, effect sizes, degrees of freedom and P value noted<br>*Give P values as exact values whenever suitable.*
☒	☐	For Bayesian analysis, information on the choice of priors and Markov chain Monte Carlo settings
☒	☐	For hierarchical and complex designs, identification of the appropriate level for tests and full reporting of outcomes
☐	☒	Estimates of effect sizes (e.g. Cohen's d, Pearson's r), indicating how they were calculated

Our web collection on statistics for biologists contains articles on many of the points above.

Software and code

Policy information about availability of computer code

Data collection	Data Collection was supervised by the CRO "KKS"
Data analysis	R (v4.3.1), FSL (https://fsl.fmrib.ox.ac.uk/)(version 1.12.3), Spinal Cord Toolbox (SCT)(version 7.0); SPM12 (UCL, London, UK)(version 7219)

For manuscripts utilizing custom algorithms or software that are central to the research but not yet described in published literature, software must be made available to editors and reviewers. We strongly encourage code deposition in a community repository (e.g. GitHub). See the Nature Portfolio guidelines for submitting code & software for further information.

Data

Policy information about [availability of data]

All manuscripts must include a [data availability statement]. This statement should provide the following information, where applicable:

- Accession codes, unique identifiers, or web links for publicly available datasets
- A description of any restrictions on data availability
- For clinical datasets or third party data, please ensure that the statement adheres to our [policy]

Source data for all figures and tables are provided with this paper. Remaining deidentified participant data underlying the results reported in this article are not publicly available due to patient privacy and ethical restrictions. However, these data will be made available to qualified investigators upon reasonable request to the corresponding author. Access is subject to approval by the Nogo Inhibition in Spinal Cord Injury steering board and will be available beginning 12 months and ending 36 months after publication.

Research involving human participants, their data, or biological material

Policy information about studies with human participants or human data. See also policy information about sex, gender (identity/presentation), and sexual orientation and race, ethnicity and racism.

Reporting on sex and gender

Sex and gender was noted as self reported.

Reporting on race, ethnicity, or other socially relevant groupings

Was not obtained as not routinely asked in the EMSCI network data collection.

Population characteristics

The following variables are collected:
• Age (years)
• Gender (male, female, intersexual/diverse, unknown)
• Year of birth
• Nodes
Age in years will be derived by subtracting year of birth from year of randomization. For analyses, age will be categorized as follows: 18-64 and ≥ 65 years old, with respect to meeting the inclusion criterion “Age 18-70“.
Medical history (Acute (spinal cord) injury related disorders, other prior diseases)
• Start/end date
• Ongoing (other prior diseases)
• General medical history (other prior diseases as free text)
• Spinal cord injury (body parts affected) (yes/no)
o Spine
o Head and face
o Chest
o Abdomen, pelvic contents
o Extremity pelvic girdle
o Other

Recruitment

The study population will consist of tetraplegic patients ranging from 18 to 70 years of age, with an acute cervical SCI classified as AIS A-D at screening. The study will be conducted in Europe and Switzerland in conjunction with the European multinational spinal cord injury trial network (EMSCI) network (www.emsci.org). All participating sites provide comprehensive care for people with SCI, from acute treatment to long-term rehabilitation. Patients from any primary acute care hospitals can be referred to any of the specialized sites, which will be part of the trial for screening and for possible study participation. On a regular basis referring hospitals will be informed and updated about the NISCI-trial.
Furthermore, the EMSCI network may involve additional backup sites that are already actively participating in the EMSCI observational study if recruitment falls behind schedule.

Ethics oversight

The NISCI trial received ethical approval from the relevant committees for each site: Cantonal Ethics Commission Zurich (Switzerland, 2016-01042), Heidelberg University Hospital (Germany, Afmu-815/2018), Fundació Unió Catalana Hospitals (Spain, 2016-001227-31), and University Hospital Motol (Czechia, EK-1367/19).
Before the start of the trial, the trial protocol, informed consent document, and any other appropriate documents will be submitted to the independent Ethics Committees (EC) as well as to the competent authorities of each participating country.
A written favorable opinion/vote of the EC and an approval by the competent authority are a prerequisite for initiation of this clinical trial. The statement of EC should contain the title of the trial, the trial code, the trial site, and a list of reviewed documents. It must mention the date on which the decision was made and must be officially signed by a committee member. This documentation must also include a list of members of the EC present on the applicable EC meeting and a GCP compliance statement.
The investigator and the Sponsor (trial master file) will keep a record of all communication with the EC and the regulatory authorities.
Before the first subject is enrolled in the trial, all ethical and legal requirements must be fulfilled.

All planned substantial amendments (e.g. trial protocol amendments) have to be signed by the sponsor and biometrician and will be submitted to EC and the competent authority for approval.

Note that full information on the approval of the study protocol must also be provided in the manuscript.

Field-specific reporting

Please select the one below that is the best fit for your research. If you are not sure, read the appropriate sections before making your selection.

☒ Life sciences ☐ Behavioural & social sciences ☐ Ecological, evolutionary & environmental sciences

For a reference copy of the document with all sections, see nature.com/documents/nr-reporting-summary-flat.pdf

Life sciences study design

All studies must disclose on these points even when the disclosure is negative.

Sample size

The power calculation is based on the mean delta changes in the EMSCI data of the control group (nodes 4, 5, 8, 9, 10, 13, 16, 17, and 18 have a mean delta UEMS of 14.3 +/-SD of 10.8 motor scores) and a 42% treatment effect (mean delta change of 20.3 motor scores). Using t-tests means with an allocation 3:1, an estimated two-sided α error probability of 0.05 and a power (1-β err prob) of 0.8 a total of about 106 patients would be required. For an adequate powering of the study, we assume that 20% of patients will drop out of follow-up, which means that at approximately 114 patients will be needed to compensate. The protocol was amended in order to get more subjects exposed to NG101. After approval of the amendment the randomization ratio will be changed from 1:1 to 3:1 (NG101:placebo). If the randomisation ratio is altered after 30 subjects have been included at 15:15 and the remaining subjects will be recruited at 63:21, the resulting number of subjects is expected to be 78:36. The power of the test is expected to go down to 66%. The power calculation based on a ttest is a reasonable (and conservative given that the variance of the estimate should be lower with explanatory variables explaining some of the variance) approximation to parameter tests from the model outlined there. The analysis of delta UEMS changes between 2 weeks- 6 months compared to 2 weeks-12 months reveals that the UEMS scores have reached about 90% of their recovery within the first 6 months after injury and late changes are rather minor. These findings allow for a total study duration of 5.5- 6 months for each patient to reveal the effectiveness of NG101.

Data exclusions

No data set was excluded, but in case of severe MR artifacts, some data points could not be considered - this detaield information can be found in the methods.

Replication

Double blinded multicentre multinational RCT

Randomization

Randomisation
After a patients' eligibility according to inclusion and exclusion criteria has been confirmed, the patient was registered at the randomisation server via https://randomizer.at.
The randomisation server provided the number of a package available at the site. Neither package number nor study medication, even if unopened, was reassigned after an erroneous randomisation.
The allocation of treatment used a balancing algorithm (Big stick allowing for an imbalance of up to 3 patients per cohort) stratified according to the cohorts obtained by the URP based stratification algorithm.
The cohorts (nodes) were derived from the screening (not baseline) measurements because the model has been developed on data obtained about 2 weeks after injury.
Included nodes:
Node 4: UEMS score ≤ 3, AIS = A
Node 5: 3 < UEMS score ≤ 11, AIS = A
Node 8: UEMS score ≤ 11, AIS > A, LEMS score = 0, light touch total score ≤ 62
Node 9: UEMS score ≤ 11, AIS > A, LEMS score = 0, light touch total score > 62
Node 10: UEMS score ≤ 11, AIS > A, LEMS score > 0
Node 13: 11 < UEMS total score ≤ 28, AIS = A or B
Node 16: 11 < UEMS score ≤ 17, AIS > B, LEMS score ≤ 17
Node 17: 17 < UEMS score ≤ 28, AIS > B, LEMS score ≤ 17
Node 18: 11 < UEMS score ≤ 28, AIS > B, LEMS score > 17
Excluded nodes:
Node 20:28 < UEMS score ≤ 38
Node 21: UEMS score > 38

Blinding

This will be a double-blind study. All patients and study site staff (excluding staff receiving IMP shipments and preparing the injections) will remain blinded to the treatment assignment. By handling and preparing IMP, the designated site staff responsible for receipt of IMP shipments and preparation of injections may become unblinded to treatment assignment and must maintain the blind and not reveal any unblinding information to other study personnel.

Reporting for specific materials, systems and methods

We require information from authors about some types of materials, experimental systems and methods used in many studies. Here, indicate whether each material, system or method listed is relevant to your study. If you are not sure if a list item applies to your research, read the appropriate section before selecting a response.

Materials & experimental systems

n/a	Involved in the study
☐	☒ Antibodies
☒	☐ Eukaryotic cell lines
☒	☐ Palaeontology and archaeology
☒	☐ Animals and other organisms
☐	☒ Clinical data
☒	☐ Dual use research of concern
☒	☐ Plants

Methods

n/a	Involved in the study
☒	☐ ChIP-seq
☒	☐ Flow cytometry
☐	☒ MRI-based neuroimaging

Antibodies

Antibodies used

Dose rationale
Clinical dose estimates for the preceding phase-I clinical trial were calculated from the results obtained in the monkey (macaque) pharmacodynamic studies, where internalization of the NG-101/Nogo-A complex is demonstrated after four weeks of continuous intrathecal infusion of 1.08 mg/day. Considering the interspecies difference in NG-101's affinity to human and monkey Nogo-A protein, the estimated effective dose in man is 5 mg/day. Since a dose of 60 mg/day is well tolerated by monkeys in the 28-day i.t. infusion toxicity study it provided the basis for estimating the safe starting dose for the completed first-in-man study in acute paraplegic SCI patients. The human dose equivalent, 300 mg/day, is based on interspecies differences in compartmental volumes (CSF volume) and antibody affinity. Division by the default safety factor of 10 would result in a maximum safe starting dose of 30 mg/day. However, this dose level is already well above the estimated effective dose in humans of 5 mg/day, which is derived from the 1 mg/day dose level used in the above mentioned proof of efficacy study in monkeys. Therefore, a starting dose of 5 mg/day has been selected for the first into man clinical trial. NG-101 in the first-in-man study (NCT00406016) was safe and well tolerated in spinal cord injured subjects at doses up to 15 mg/day for a maximum of 28 days using continuous i.t. infusion and at doses up to 6 × 45 mg over four weeks using repeated i.t. bolus injection. Repeated i.t. bolus injections appeared to be safer and less prone to technical complications compared to the continuous infusion mode of administration, and appear to meet tolerability and pharmacokinetics expectations. Based on the data from the first-in-man study (NCT00406016), the treatment regimen in the phase II study in spinal cord injury tetraplegic patients will be repeated i.t. bolus injection of 6 × 45 mg NG-101 over four weeks.

Validation

Pharmacokinetics
CSF ELISA for NG101
For the detection of NG101 in the CSF a murine type 2 anti-idiotypic monoclonal antibody against NG101 (clone 1D2, Agro-Bio, La Ferté Saint-Aubin, France) was developed. The antibody was labeled with biotin or HRP, respectively. 0.25 µg/ml of 1D2biotin mouse anti-NG101 capture antibody dissolved in 1% BSA (Sigma) in PBS-0.1% Tween20 (Sigma) was bound on pre-coated neutravidin plates (Thermo Fisher #15507). Each plate contained a serial dilution of NG101 as internal standard and CSF samples 5- and 10-fold diluted. Detection was performed with a second monoclonal mouse anti-NG101 HRP antibody (clone 1D2 HRP). The plates were developed with TMB substrate (Pierce) and stopped with 1M HCl. The readouts were acquired on a Tecan Sparc plate reader at 450nm with 620nm correction. The ELISA had the following detection limits: LLOD <4.15 ng/ml, LLOQ 14.6 ng/ml and ULOQ 1000 ng/ml, (Precision (%CV)=25% and Accuracy (%RE)=25%).
Serum ELISA
For the detection of NG101 in serum, 2 µg/ml of a synthetic peptide (16 aa of Nogo-A corresponding to the NG101 epitope, biotin-labelled; JPT Peptide Technologies, Berlin, Germany) in PBS (Gibco) were coated on a 96 well costar plate (Corning #3690) for 2 h at 37°C. Plates were washed three times with TBS-0.1% Tween20 and blocked with SeraSub (CST Technologies) for 1.5 h at 37°C. Each plate contained a serial dilution of NG101 as internal standard. Serum samples were diluted in the blocking solution 5-fold and incubated on the plates for 2 h at 37°. Plates were then washed three times with TBS-0.1%Tween20 and incubated with a mouse anti-human IgG4 antibody (Bio-Rad #919001) diluted 1000-fold in SeraSub for 1 h at 37°C. Plates were again washed three times and incubated with a goat anti-mouse HRP-coupled antibody (Invitrogen) diluted 40,000-fold in SeraSub for 1 h at 37°C. Finally, the plates were washed six times and developed with TMB substrate (Pierce).

The readouts were acquired on a Tecan Sparc plate reader at 450 nm and 620 nm correction. The ELISA had the following detection limits: LLOD <0.244 ng/ml, LLOQ 1.7 ng/ml and ULOQ 1000 ng/ml (Precision (%CV) =25% and Accuracy (%RE) =25%).

Clinical data

Policy information about clinical studies

All manuscripts should comply with the ICMJE guidelines for publication of clinical research and a completed CONSORT checklist must be included with all submissions.

Clinical trial registration	NCT03935321
Study protocol	Attached to the submission
Data collection	May 2019 to July 2022

Outcomes

Primary objective:
To evaluate efficacy of acute treatment (initiation of drug treatment within 4 - 28 days post-injury) with NG-101 by repeated intrathecal (i.t.) bolus injections on day 168.
Primary efficacy endpoint:
Upper extremity motor scores (UEMS) according to the International Standards for the Neurological Classification of Spinal Cord Injury (ISNCSCI)
2.2 Secondary Objectives and Secondary Endpoints
Secondary objectives and endpoints:
• Effect on motor and sensory function according to the ISNCSCI protocol (ASIA impairment scale, ASIA lower extremities motor score (LEMS) and sensory scores (light touch (LT), pin prick (PP)) on day 168.
• Effect on autonomic dysfunction (i.e. bladder function as measured by bladder diary, Qualiveen questionnaire, bladder function assessment on day 168.
• Effect on functioning evaluated by the Spinal Cord Independence Measure (SCIM-III) on day 168.
• Effect on hand/upper limb function as assessed by the Graded and Redefined Assessment of Strength, Sensibility and Prehension (GRASSP) subscales on day 168.
• Effect on the Walking Index for Spinal Cord Injury (WISCI), 10-meter walk test (10mWT) and the 6-minute walking test (6MWT) on day 168.
• Effect on neurophysiological parameters (nerve conducting velocity, Somatosensory evoked potentials) on day 168.
• To evaluate the pharmacokinetics (PK) and immunogenicity of NG-101. All PK/ IG samples collected from day 0 until day 84 will be included in the respective PK and immunological response analyses.

T4.3 Characterization of the initial patients conditions based on the morphology of the lesion: serial state-of-the-art clinical imaging protocols Mo 01-54
The immediate (i.e. core) lesion area will be surrounded by tissue that is less severely affected (called penumbra) and eventually goes over into totally intact cord tissue. We will use the clinical T1 and T2 weighted images to characterize the initial state of the lesion (these measures might be to some extent affected by metal artifacts due to surgery) by assessing the lesion length, shape and volume, compression ratio, intramedullary cyst formation and edema (Miyanji et al., 2007). We will then assess the evolution of the focal injury zone (from the core of damage to the penumbra and eventual normal cord tissue) to discern different levels of cord damage (i.e. a gradient from normal to severely damaged tissue). Crucially, we will assess whether the anti-Nogo-A antibody treatment induced effects will occur close to or across the lesion site and is more effective in those patients with more preserved tissue.
UZH will lead on the development of a combined analysis pipeline incorporating the clinical and advanced MRI outcome measures. MPG will contribute novel post-processing methods developed in T4.2.
Partners: UZH, MPG and all participating SCI centers
T4.4 Assessment of the responsiveness of the neuroimaging biomarkers beyond the lesion area: serial quantitative MRI of microstructure Mo 24-60
Quantitative markers of the spinal cord above the level of lesion (at level C1/2) and at the brain will be assessed in anti-Nogo-A antibody treated and control treated patients. We use Jim 7·0 (Xinapse systems, Aldwincle, UK) for the measurement of cross-sectional grey and white matter area of the cervical cord (Horsfield et al., 2010). We use tensor based morphometry (Ashburner and Ridgway, 2012), as implemented in SPM12 (MPG), to estimate regional changes of brain volume over time for anti-Nogo-A antibody treated patients and control treated patients. We use the advanced FLASH protocol from task T4.1 to calculate the myelin sensitive quantitative parameter maps of MT saturation and apparent longitudinal relaxation rate R1 (defined as 1/T1) to assess microstructural changes over time. Results of patient analyses will be compared to equally processed rat spinal cords which are available in the UZH laboratory and for which histological analyses will be done to demonstrate tract de- and regeneration, inflammatory events and myelin loss and reformation.
Statistical differences in their MRI advanced outcome measures of the spinal cord and brain (Figure A) between the two groups will be assessed with multiple linear regression analysis including confounds such as age and gender (Freund et al., 2013). Interaction terms will also be added to assess between-subject-type differences. To model sensitivity to treatment effects, we will be using models with clinical variables as outcomes and imaging measurements as predictors. We will be introducing interaction terms to assess whether anti-Nogo-A antibody treatment is an independent predictor of a certain clinical status. Where multiple variables are thought to represent similar underlying pathological processes (e.g. R1 and the MT parameter) latent variables will be used to assist interpretation and reduce multiple testing.

University of Zürich will lead on the selection of the most sensitive and accurate neuroimaging biomarkers that are most likely to be successful in detecting treatments effects for later clinical trial phases

Safety objectives and endpoints:
To evaluate the safety of acute treatment (initiation of drug treatment within 4 - 28 days post--injury) with NG-101 by repeated intrathecal bolus injections (6 injections of 45 mg each over 4 weeks)
Safety endpoints:
• Adverse Events (Frequency, type, duration and intensity of AEs and SAEs)
• Relationship of AE/SAE frequency and time and duration of study medication administration
• Documented reasons for any unplanned study medication interruptions and/or withdrawal from the study
• Vital signs (blood pressure, heart frequency, body temperature)
• Muscle spasticity measured by the Modified Ashworth Scale
• Effect on pain (neuropathic pain and non-neuropathic pain) assessed by SCI pain data set, allodynia questionnaire & SCIPI

Plants

Seed stocks

Report on the source of all seed stocks or other plant material used. If applicable, state the seed stock centre and catalogue number. If plant specimens were collected from the field, describe the collection location, date and sampling procedures.

Novel plant genotypes

Describe the methods by which all novel plant genotypes were produced. This includes those generated by transgenic approaches, gene editing, chemical/radiation-based mutagenesis and hybridization. For transgenic lines, describe the transformation method, the number of independent lines analyzed and the generation upon which experiments were performed. For gene-edited lines, describe the editor used, the endogenous sequence targeted for editing, the targeting guide RNA sequence (if applicable) and how the editor was applied.

Authentication

Describe any authentication procedures for each seed stock used or novel genotype generated. Describe any experiments used to assess the effect of a mutation and, where applicable, how potential secondary effects (e.g. second site T-DNA insertions, mosiacism, off-target gene editing) were examined.

Magnetic resonance imaging

Experimental design

Design type

structural MRI data of the spinal cord and brain was acquired. In this paper we assess only the spinal cord.

Design specifications

NaN

Behavioral performance measures

NaN

Acquisition

Imaging type(s)

Structural

Field strength

The following scanner were used at the different sites: a 1.5 T Toshiba scanner (Canon Medical Systems Cooperation, Otawara, Tochigi, Japan) in Hessisch-Lichtenau, in 3T Philips scanner (Philips Healthcare, Best, The Netherlands) in Murnau, Nottwil, Halle and Berlin, a 1.5T Siemens scanner (Siemens Healthcare, Erlangen, Germany) in Barcelona and Prague or a 3T Siemens scanner (Siemens Healthcare, Erlangen, Germany) in Zurich Barcelona, Bayreuth, Basel, Bochum, and Heidelberg. Throughout the assessment process, raters were blinded to treatment arms and time points.

Sequence & imaging parameters

T1 and T2weigthed axial and sagittal scan in the cervical cord, MPM protocol with T1, PD and MTsat- weighted echoes

Area of acquisition

Brain and cervical spinal cord

Diffusion MRI ☐ Used ☒ Not used

Preprocessing

Preprocessing software

SPM, FSL, Spinal Cord Toolbox, R software

Normalization

If data were normalized/standardized, describe the approach(es): specify linear or non-linear and define image types used for transformation OR indicate that data were not normalized and explain rationale for lack of normalization.

Normalization template

PAM50 from the SCT

Noise and artifact removal

Lesion volume and midsagittal tissue bridges (TB) width were quantified from sagittal T2-weighted MRI scans.24 The

midsagittal slice was defined as the central slice at the injury epicenter showing the maximal extent of preserved tissue. Manual delineation of lesion boundaries was based on T2w hyperintensity and was done on each sagittal slice using JIM7 (Xinapse Systems) by an operator blinded to the timing of the MRI scan and treatment group (Supplementary Material Figure 7). Lesion volumes were calculated by summing the lesion areas across slices and multiplying by the slice thickness (2 - 4.4mm). Corticospinal tract (CST) and dorsal column (DC) damage was assessed by determining the overlap between segmented lesion volume and identified tracts in the axial plane. Participants were grouped by TB (≥ 1.0mm vs < 1.0mm), with the cutoff derived from analyses of a multicenter cohort using a URP model of 3 month UEMS outcomes.7

Quantitative myelin-sensitive imaging was performed using a Multiparameter Mapping (MPM) protocol that included magnetization transfer saturation (MTsat) weighted, proton density-weighted, and T1-weighted scans. Scanner harmonization was ensured by following a standardized multicenter protocol, previously validated,10,22 which demonstrated consistent parameter estimates in the cervical spinal cord across seven scanners, with intra- and inter-site coefficients of variation ranging from 2.5-12% for MT, R1, and PD, and 1.1-4.0% for morphometric measures. These images were used to compute quantitative maps of MTsat via the hMRI toolbox25 embedded in SPM12 (UCL, London, UK), which applies corrections based on separately acquired $B1^+$ and $B1^-$ maps to account for transmit and receive field inhomogeneities. Due to poor image quality at C3 across the imaged cohort, analysis focused on the C1-C2 levels, which was above the lesion level for all participants. Processing was performed using the Spinal Cord Toolbox (SCT)26 with an automated pipeline for registration, warping, and the extraction of both morphometric and microstructural parameters. Spinal cord segmentation was performed using the sct_deepseg function, and the resulting masks were visually inspected and manually corrected when needed using FSL (https://fsl.fmrib.ox.ac.uk/). The corrected masks were registered to the PAM50 template27 using a combination of affine and nonlinear transformations, and reverse deformation fields were applied to warp the white matter (WM) and gray matter (GM) atlases into subject space. To mitigate partial volume effects, cross-sectional area (CSA) and tract-specific metrics were extracted using SCT's atlas-based weighted masks and maximum a posteriori estimation, which account for mixed tissue contributions at voxel boundaries.

Volume censoring

NaN

Statistical modeling & inference

Model type and settings

All statistical analyses were conducted using R (v4.3.1). All frequentist inferential statistics are reported as the test statistic with corresponding degrees of freedom, p-values, effect size statistics, and 95% confidence intervals (CI) of the difference. Between-group differences at the screening visit were tested using independent-sample t-tests using the t.test() function in R. Longitudinal changes in MRI biomarkers were assessed using linear mixed-effects models using the lmer() function of the lme4() package in R. Fixed effects included treatment group (NG101 vs. placebo), time (days post baseline), their interaction (time × treatment), time to treatment initiation, age, sex, AIS, NLI, and the baseline value of the outcome measure. Center was modeled as a random intercept to account for site-related differences when model convergence was achieved; otherwise, it was included as a fixed effect. Subject-specific random slopes for time were included to capture within-subject changes over time. The estimated rates of change for each group reflect the average linear change in the outcome over the entire follow-up period (baseline to 6 months). Group differences were assessed through the interaction between treatment group and time. Stratification strategies were evaluated based on: 1) clinical stratification: motor-complete vs. motor-incomplete, 2) electrophysiological biomarkers: 'preserved' vs. 'absent' tibial SSEP or C8 dSSEP, 3) MRI-based biomarkers: TB ≥ 1.0mm vs. < 1.0mm, 4) combined stratification: integration of preserved TB (≥ 1.0mm) and preserved tibial SSEP or C8 dSSEP.

Stratification findings were based exclusively on data acquired at the screening visit. For every stratification strategy, longitudinal changes in a) UEMS and b) SCIM self-care were assessed using linear mixed-effects models using the lmer() function of the lme4() package in R with treatment group (NG101 vs. placebo) and time as fixed effects and a random intercept for each patient to account for within-subject variability. Group differences were assessed through the interaction between treatment group and time.

Effect(s) tested

NaN

Specify type of analysis: ☐ Whole brain ☐ ROI-based ☐ Both

Statistic type for inference

All statistics of extracted MRI markers was performed in R

(See Eklund et al. 2016)

Correction

Describe the type of correction and how it is obtained for multiple comparisons (e.g. FWE, FDR, permutation or Monte Carlo).

Models & analysis

n/a	Involved in the study
☒	☐ Functional and/or effective connectivity
☒	☐ Graph analysis
☐	☒ Multivariate modeling or predictive analysis

Multivariate modeling and predictive analysis

Independent variables: Age, sex, level of injury, AIS grade, baseline value of outcome measure
